# Respecting gender diversity in academic writing

**DOI:** 10.1080/10872981.2023.2169921

**Published:** 2023-01-18

**Authors:** Jame A. Agapoff, Gerrit I. van Schalkwyk

**Affiliations:** a Department of Psychiatry, University of Hawai‘i at Mānoa, Honolulu, Hawai‘i, USA; b Intermountain Health, Salt Lake City, Utah, USA

To the Editor:

Academic medicine has a long history of supporting the scholarship of minority providers and researchers. Still, many gender diverse persons remain invisible and marginalized in academia.

Historically, minority groups have been excluded from research impacting their communities. Some have even argued minority voices are “too biased” to be included. This systemized exclusion leads to pathologizing language, opinions, and guidelines that act to further perpetuate stereotypes and prejudices toward minorities. A group that has suffered immensely from this type of exclusion is the transgender community [1].

Until 2013, the identities of gender diverse people were categorized as mental disorders. In the United States, access to gender affirmative care still requires a diagnosis of gender dysphoria [2]. Even with improved guidelines [3], many transgender persons still experience dehumanizing diagnostic and assessment periods to access care [4].

Lack of visibility and representation of gender diversity is a significant problem in academic medicine and research. Some surveys designed to assess diversity in science often ask about sex and not gender [5], excluding (and rendering invisible) many who identify as anything outside of the traditional gender binary.

Visibility is the first step to inclusion.

Academic medicine can show respect for gender diversity and create greater inclusion and visibility by taking the following concrete steps:

- Giving authors the opportunity to list their pronouns on published manuscripts
- Ensuring that reviewer, author, and editor profiles include options for gender identity that are non-binary, and again offer the opportunity to identity pronouns
- Work towards normalization of gender not being assumed by avoiding gendered language in correspondence between authors, reviewers, and editors

We need to stop blaming programs and computer systems for our lack of inclusion. As an academic and research community we need to do better.
